# The NSPCC UK Minding the Baby® (MTB) home-visiting programme, supporting young mothers (aged 14–25) in the first 2 years of their baby's life: study protocol for a randomised controlled trial

**DOI:** 10.1186/s13063-016-1618-4

**Published:** 2016-10-07

**Authors:** Elena Longhi, Lynne Murray, Rachael Hunter, David Wellsted, Samantha Taylor-Colls, Kathryn MacKenzie, Gwynne Rayns, Richard Cotmore, Peter Fonagy, Richard M. Pasco Fearon

**Affiliations:** 1 Research Department of Clinical, Educational and Health Psychology, University College London, Gower Street, London, WC1E 6BT UK; 2 School of Psychology and Clinical Language Sciences, University of Reading, Harry Pitt Building, Early Gate, Reading, RG6 7BE UK; 3 Priment Clinical Trials Unit, Research Department of Primary Care and Population Health, UCL, Royal Free Campus, London, NW3 2PF UK; 4 University of Hertfordshire, Hatfield, AL10 9AB UK; 5 The Anna Freud Centre, 21 Maresfield Gardens, London, NW3 5SD UK; 6 National Society for the Prevention of Cruelty to Children (NSPCC), Weston House, 42 Curtain Road, London, EC2A 3NH UK; 7 Department of Psychology, Stellenbosch University, Private Bag X1, Matieland, Stellenbosch, 7602 South Africa

**Keywords:** Minding the Baby®, Home-visiting programme, First-time mothers, Attachment, Reflective functioning

## Abstract

**Background:**

Young mothers living in low-income urban settings often are exposed to significant and chronic environmental difficulties including poverty, social isolation and poor education and typically also have to cope with personal histories of abuse and depression. Minding the Baby® (MTB) is an interdisciplinary home-visiting programme developed to support first-time young mothers, which integrates primary care and mental health approaches into a single intensive intervention from the last trimester of pregnancy until the child’s second birthday. The primary aim of the intervention is to promote caregiver sensitivity, and, secondarily, to promote both child and maternal socioemotional outcomes.

**Methods/design:**

This is a multisite randomised controlled trial (RCT) with a target recruitment of 200 first-time adolescent mothers (under 26 years of age). One hundred participants will be randomised to the MTB group and they will receive the MTB programme in addition to the usual services available in their areas. Those participants not allocated to MTB will receive Treatment as Usual (TAU) only. Researchers will carry out blind assessments at baseline (before the birth of the baby), and outcome assessments around the child’s first and second birthdays. The primary outcome will be the quality of maternal sensitivity and the secondary outcomes will focus on attachment security, child cognitive/language development, behavioural problems, postponed childbearing, maternal mental health and incidents of child protection interventions.

**Discussion:**

This study evaluates the Minding the Baby® programme in the UK. In particular, this RCT explores the effectiveness of this integrative approach, which focusses on maternal mental issues as well as parent-infant interaction, parental concerns and developmental outcomes.

**Trial registration:**

ISRCTN08678682 (date of registration 3 April 2014).

**Electronic supplementary material:**

The online version of this article (doi:10.1186/s13063-016-1618-4) contains supplementary material, which is available to authorized users.

## Background

### Overview and rationale

The NSPCC, in collaboration with University College London, the University of Reading, the Yale Child Study Centre and the Yale School of Nursing, is initiating a multisite study of the effectiveness of a targeted prevention programme that incorporates well-established principles of home visiting with a more comprehensive package of care for the developing mother-infant relationship. The programme represents an important opportunity to advance the UK’s provision of evidence-based support for at-risk families and to intervene effectively in the intergenerational cycle of disadvantage. The Minding the Baby® (MTB) programme is an interdisciplinary intervention that was developed and tested by a team of researchers and clinicians at the Yale Child Study Centre and the Yale School of Nursing [1]. MTB combines many of the benefits of home-visiting programmes – particularly their relative cost-effectiveness, client acceptability and accessibility – with a coherent, evidence-based clinical dimension that is informed by, and directly targets, well-studied mechanisms of risk in early child development. In focussing on key domains of parent-child relationships where disturbances are known risk factors for later child maladjustment, particularly the sensitivity of parental care, the security of infant-parent attachment and the parent’s capacity to reflect on the child as an autonomous agent with needs, feelings and thoughts, the programme aims to combine best clinical practice in early prevention with scientific evidence regarding the developmental processes that promote optimal child outcomes. Currently, the UK health and social care systems offer a range of services to young families targeting mental health or promoting family relationships from birth, which are not always evidence-based and vary considerably from region to region. Home-visiting programmes are characterised by the presence of consistent and reliable support figures with high-quality training who are capable of addressing a broad range of parenting concerns from the practical to the emotional [2]. The highly influential Nurse Family Partnership (NFP) model is a well-known example that has been found to be effective for several important early child and maternal outcomes [3]. A notable limitation of existing home-visitation programmes, however, is the relative lack of focus on supporting parent-child interaction and particularly attachment. This is a central target of MTB [4, 5]. Longitudinal outcome studies clearly show that disturbances in the quality of care can have lasting negative consequences for children’s development, and the long-term social and financial costs associated with these poor outcomes are likely to be considerable [6]. The potential value of effective early intervention focussed on sensitivity of care, particularly for parents experiencing multiple social adversities, therefore, cannot be overstated.

This randomised clinical trial will test the hypothesis that an intensive home-visiting programme focussed on promoting young parents’ sensitive attunement to their infants and their ability to mentalise on their baby’s thoughts, feelings and needs, will lead to improvements in the sensitivity of parenting of children age 2 years compared to parents who receive routine care. The study will also examine several secondary hypotheses, including that the programme will increase offspring rates of secure attachment, improve cognitive and behavioural outcomes and promote maternal mental health.

### Background and significance

Although rates of teenage pregnancies have been dropping in the UK over the last 10 years, it remains the case that such pregnancies are greatly over-represented in low-income urban populations [7]. The many environmental stressors that these young parents face (poverty, single parenthood, social isolation and poor educational achievement [8]) are often amplified by personal histories of abuse, depression and posttraumatic stress disorder (PTSD) [9, 10]. These parents may find themselves not only having to deal with their own developmental needs but also trying to take on the complex roles and responsibilities of parenting. It is perhaps not surprising that young parents living in these circumstances are more susceptible to mental health problems and may struggle to become responsive nurturing parents [11, 12]. Social disadvantage more generally represents a broad but very reliable marker of a host of contextual, psychological and developmental risk factors that have well-established negative impacts on the quality of parenting and on child development [13–15]. The MTB programme is aimed at supporting young parents facing multiple social stressors, and raising their first infant in adverse social circumstances, in order to promote positive parenting, raise rates of secure attachment and improve child developmental outcomes.

The MTB programme is the result of an interdisciplinary collaboration between the Yale School of Nursing and the Yale Child Study Centre. MTB is an intensive and preventive home-visitation programme for young first-time parents. MTB primarily evolved from two home-visiting models that originated in the US; the NFP and the infant-parent psychotherapy model. David Olds and colleagues developed the NFP programme [3], which involves home visits by highly trained nurses to vulnerable high-risk first-time mothers. Home visits begin at the end of the second trimester of pregnancy and continue through the child’s second birthday. Extensive research on the effectiveness of the NFP programme in high-risk populations showed improved health, parenting and developmental outcomes [2, 3, 16–23]. A different emphasise is on the infant-parent psychotherapy model which was developed to protect infants and help parents with mental health problems, often as a result of ongoing trauma. Although this model has been less rigorously tested than the NFP programme, positive child outcomes were found. In particular, this programme appears to supports the development of healthy mother-child relationships and secure attachment [24], both of which are prognostic indicators of longer-term positive developmental outcomes in the child [25].

The MTB programme brings together both these models, providing a holistic intervention that not only addresses maternal mental health issues but also health, attachment and life course outcomes for mother, child and family. Thus, MTB aims to bring together health, developmental, attachment and mental health approaches. By incorporating both nursing and mental health approaches, MTB serves to address some of the more complex needs of mothers and families at risk.

### Attachment and Reflective Functioning

It is firmly established in the attachment field that the quality of the infant’s attachment to their primary caregiver is robustly related to a range of child outcomes [5]. MTB builds on this evidence and makes the promotion of secure attachment a primary clinical objective as a means of bringing about positive changes in the infant’s social, emotional and cognitive development. Originally, Ainsworth and colleagues [26] suggested that a mother’s ability to respond sensitively to her child’s cues would be crucial for the development of secure mother-infant attachment. Later research [27] empirically tested this hypothesis and found broad support for the role of sensitivity in secure attachment. Furthermore, recent work has highlighted the role of the mother’s own mental state with respect to attachment – referred to as her internal working model (IWM) of attachment, in shaping the sensitivity of care, and thus her child’s attachment security [28]. These attachment representations are thought to shape how a parent perceives their child and, accordingly, how they respond to the child’s behaviour, cues and communications [29].

A critical feature of the way in which parents think about their children is their ability to consider the child’s thoughts, feelings and beliefs, and to treat the child, therefore, as an individual with a mind. Crucially, research indicates that this ability not only to think of the child as an individual with their own thoughts and feelings, but also to understand and make a causal connection between the child’s behaviours and their underlying feelings and experiences, is crucial in the development of a secure attachment [30]. This capacity has been termed by Fonagy and colleagues as ‘mentalisation’ or ‘Reflective Functioning’ (RF) [31]. Slade and colleagues’ research in this area has demonstrated consistent relationships between a mother’s ability to mentalise, maternal behaviour, and child attachment [30, 32].

The MTB programme is rooted in this developmental theory and, at its core, the MTB programme aims to increase the parent’s capacity to think about their child and reflect upon their thoughts, and feelings, and to respond in a sensitive and attuned way to the child’s cues and communications.

### Minding the Baby®: an interdisciplinary approach

The home-visiting intervention programmes presented above have mostly focussed on either the practical aspects of parenting or the quality of the mother-child relationship and attachment. MTB aims to address both these elements of parenting.

The UK MTB clinical team includes two qualified practitioners: a nurse or health visitor and a social worker who are both highly trained and supervised in particular techniques and developmental approaches tailored for working with vulnerable young mothers. The nurse provides advanced levels of practical parenting support including individual and family health assessments, nutritional advice and family planning. The social worker provides mental health support to mother and baby, in-home assessment and intervention for mild to moderate mental health problems like depression, anxiety and PTSD symptoms that the mother might be affected by. Crucial to the success of the MTB programme is the mother’s relationship with the MTB practitioners. Their engagement and fostering of ongoing relationships with these at-risk, first-time young mothers, as well as having the professional expertise that matches their complex health, social and mental health needs, is aimed to diminish attrition from the programme. This kind of integrative model is considered to be crucial for maximising both parental and child outcomes across a range of domains.

Following the Yale model, the UK MTB is grounded in well-established developmental research, builds on the experience of similar successful programmes, is a relationship-based model, delivers a flexible model of care design to match the varying and often complex needs of at-risk families, and has a robust, manualised system of training and supervision.

### Aims and objectives

Aim 1: the primary aim of this study is to test whether participation in the MTB programme leads to improvements in the quality of parenting and specifically the degree of maternal sensitivity.

Aim 2: the secondary aims of the study are to measure the effects of the MTB programme in relation to (1) maternal outcomes including maternal mental health, maternal RF and postponed subsequent child bearing and (2) infant outcomes including incidents of child protection intervention, attachment security to the parent, cognitive and language development and behavioural problems.

Aim 3: a further key secondary aim is to assess the cost benefit/cost-effectiveness of the MTB programme in order to sustain future programmes.

## Methods

### Design

**Fig. 1:**
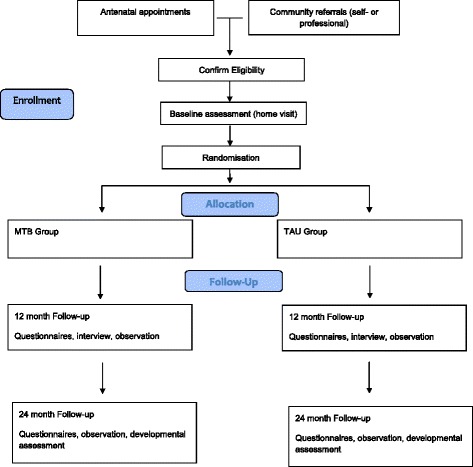
Flow diagram of the study design

This is a multisite randomised controlled trial, with randomisation at the case level. This trial will utilise a two-arm design, with random allocation to either MTB plus Treatment as Usual (TAU) or a TAU-only control condition. Allocation will be by minimisation, controlling for maternal age, maternal depression and study site. Figure 1 shows a flow diagram of the study design, and the Standard Protocol Items: Interventions for Reporting Trials (SPIRIT) checklist is presented in Additional file 1 [33, 34].

### Outcome measures

#### Primary outcome

The primary outcome is the quality of parenting, operationalised as maternal sensitivity [26]. Maternal sensitivity will be measured at ages 1 and 2 years and will be treated as a continuous score, with both time points included in the primary analysis.

In order to measure parenting sensitivity at ages 1 and 2 years, we will use several short tasks from our existing studies of attachment and another ongoing clinical trial. The first task focusses on mother-infant interaction in the context of free-play. Known as the ‘three-boxes procedure’, the mother shows the child experimenter-provided toys in three containers in a set order [35–37]. The second is a procedure pioneered by Smith and Pederson [36]. In this task, mother and infant are left to explore a relatively empty room, while the mother must also complete a distraction questionnaire. Another task involves brief observations, one focussing on book-sharing and the other on a difficult-to-manipulate toy. Finally, we are using a separate joint book-reading observation in which the content of the book involves strong attachment-related scenarios. In each case, maternal sensitivity will be rated, using Emotional Availability Scales [38], an observation tool. The scales describe and assess four dimensions on the adult side (sensitivity, structuring, nonintrusiveness and nonhostility) and two dimensions on the child side (responsiveness and involvement with the caregiver). Dimensions are measured on a scale, with scores of between 1 and 7. Scales will be standardised and summed to yield a total score across all tasks for the main analysis. The use of specific contexts for mother-infant interactions will also allow us to determine whether the intervention is changing the particular processes associated with each domain of child development in tertiary analyses.

#### Secondary outcomes

*Child attachment security*, measured with the Attachment Q-Set (AQS: [39]), which will be administered at year 2. The AQS is based on a set period of observation of children aged 1–5 years in the home environment. The AQS consists of a set of 90 cards with a specific behavioural characteristic described on each card that is age-appropriate. The cards are used as a standard vocabulary to describe the behaviour of a child in a home setting, with an emphasis on secure-base behaviour. The researcher who has observed the parent and child ranks the cards into several piles from ‘most descriptive of the subject’ to ‘least descriptive of the subject’. The Q-Set provides a score along a continuum of secure to insecure. The Q-Set has shown good convergent and discriminate validity [40] and is a strong predictor of later developmental outcomes [41].

*Child cognitive and language development* will be assessed at year 2 using the Bayley Scales of Infant and Toddler Development, third edition (Bayley-III) [42]. The Bayley-III is an individually administered assessment that evaluates the child’s mental and motor development. The scales are administered when children are between the ages of 2 months and 42 months. This yields two separate continuous scales representing overall cognitive development and language development. The Bayley-III is a standardised instrument and the cognitive scales and language composite correlate, respectively (*r* = .79) and (*r* = .82), with the WPPSI-III Full-scale IQ (Weschler Preschool and Primary Scale of Intelligence), reported for children aged 28–42 months. Bayley-III is also UK-validated [43].

*Behavioural problems* will be assessed with the Child Behaviour Checklist (CBCL: [44]) questionnaire at year 2. This consists of a 100-item parent-report questionnaire and is valid for children from 18 months and older. The CBCL measure yields three age-normed scales of Internalising Problems (i.e. anxious, depressive, and over-controlled), Externalising Problems (i.e. aggressive, hyperactive, noncompliant, and under-controlled) and Total Problems. Parents record responses with: 0 (Not true, as far as I know), 1 (Somewhat or Sometimes true) or 2 (Very true or Often true). The analysis will focus on the Total Problem scale. The CBCL is one of the most widely-used standardised measures in child psychology for evaluating maladaptive behavioural and emotional problems [45].

*Postponed child-bearing* will be assessed at each follow-up when mothers will be asked about their pregnancy status. The number of months from baseline to the next pregnancy will be used for analysis.

*Maternal mental health* will be measured with the Edinburgh Postnatal Depression Scale (EPDS: [46]) questionnaire which will be administered at baseline, year 1 and year 2. The EPDS is a 10-item questionnaire screening for postnatal depression. Mothers’ responses are on a scale of 0 to 3, and a score is calculated adding individual items. All three total scores from the questionnaire will be entered into the analysis, with change from baseline being the outcome of interest. EPDS is a well-validated measure of depression [47] that may be used within 8 weeks postpartum but has also been applied for depression screening during pregnancy [48].

*Child Quality of Life* will be assessed at year-1 and year-2 follow-ups with the Warwick Child Health and Morbidity Profile (WCHMP) questionnaire [49]. This consists in a 10-item survey where parents are asked to report on health and morbidity in infancy and childhood. The WCHMP has shown to be reliable and valid with low interobserver variation [49]. An incremental cost-effectiveness ratio (ICER) will be calculated and the two groups of mothers compared.

*Health and social care resource use* will be collected throughout the study using the Service Use and Supports Questionnaire (SUS) [50]. This is a self-report questionnaire administered at baseline and every subsequent follow-up, i.e. 6 months, year 1, 18 months and year 2. Mothers are asked to note whether they had any input from professionals and voluntary agencies in the previous 6 months, in four areas: (1) health services, (2) mental health services, (3) support services and (4) child care services. Parents are also asked to note down the single most helpful service they have accessed over the previous 6 months. Costs are applied to service use at each time point. Total costs per patient will be calculated from the total across all follow-up points and adjusted for by baseline values. Unit costs will be obtained from the Personal Social Services Research Unit’s (PSSRU) nationally published reference costs and published studies.

#### Additional outcome measures

*Infant Behaviour Questionnaire Revised* (IBQ-R: [51]) is a parent-report questionnaire that ask parents to rate the frequency of specific temperament-related behaviours observed over the past week (or sometimes 2 weeks). The IBQ-R assesses the child’s temperament on six dimensions including activity level, soothability, fear, smiling and approach behaviours. Parents rate the frequency of specific temperament-related behaviours on a scale of 1 to 7. The IBQ-R has demonstrated good internal consistency, reliability and convergent validity [52]. The IBQ-R will be administered at year 1.

*Infant Health Outcome* data will be collected at the end of the study through a review of the infant’s/toddler’s health records. Data will be collected on birth outcomes, routine hospital visits, completeness of immunisations, accident and emergency (A&E) visits, presence of chronic health problems and number of referrals to Social Services. Unit costs will be applied to calculate the cost per infant.

*Maternal Sense of Mastery* is measured by the Pearlin and Schooler 7-item scale. Women are asked to measure the degree to which they perceive they can control their life’s chances [53]. Responses are based on a 7-item scale (agreement to disagreement), and higher scores reflect greater level of mastery. This scale has been used extensively with similar samples of young women [54]. It will be administered at baseline, 1- and 2-year follow-ups.

*Norbeck Social Support Questionnaire* (NSSQ: [55, 56]) measures multiple functional dimensions of social support: (1) affect, (2) affirmation and (3) aid. Participants are instructed to list first names or initials for each significant person in their lives who provides personal support to them. Participants are asked to identify their relationship with the individual and finally to use a 5-point rating scale to describe the amount of support available from each person. The NSSQ has shown to be a valid and reliable measure of all three functional types of social support as well as total network support [57]. It will be administered at baseline, 1- and 2-year follow-ups.

*Parent Development Interview – Revised* (PDI: Slade A, Aber JL, Bresgi I, Berger B, Kaplan M. The Parent Development Interview - Revised. The City University of New York, 2004. Unpublished Manuscript.) is a 20-question interview that assesses parents’ representations of their child, their relationships with them, and particularly their capacity to reflect on their child’s mental states. Transcribed interviews are scored for RF. Initial studies testing the validity of this measure have linked it to adult attachment, child attachment and parental behaviour both in normal and drug-using samples [4, 29, 32, 58–60]. RF is scored on a scale of 1–9 with higher scores indicating greater levels of RF. It will be administered at year 1. 

*Parenting Stress Inventory* (PSI) *Short Form* [61] is a 36-item questionnaire that measures stress levels experienced within the parenting role. Rated on a 5-point scale (agreement to disagreement), the measure contains three subscales pertaining to parenting stress. The PSI Short Form (PSI-SF) subscales have demonstrated concurrent validity with the full-length PSI [62]. The PSI-SF will be administered at baseline, 1- and 2-year follow-ups.

*PTSD Checklist-Civilian* (PCL-5: [63]). This is a 20-item PTSD screen that is closely based on the *Diagnostic and Statistical Manual of Mental Disorders, fifth edition* (DSM-V) criteria for PTSD. Participants rate each item from 0 (not at all) to 4 (extremely) to indicate the degree to which they have been bothered by the index symptom in the past month. The PCL-C has shown good psychometric properties, high rates of internal consistency, test-retest reliability and is highly correlated with other measures of trauma symptoms [64]. It will be administered at baseline, 1- and 2-year follow-ups.

*State-Trait Anxiety Inventory* (STAI: [65]) is a 40-item questionnaire that uses a 4-point Likert scale to address both state and trait anxiety. The construct and concurrent validity of the measure has been robustly demonstrated [65, 66]. It will be administered at baseline, 1- and 2-year follow-ups.

*Adult quality of life (QoL) –* The EuroQol EQ-5D 3 level (EQ-5D-3 L) is a health-related questionnaire assessing the quality of life through five dimensions (mobility, self-care, usual activities, pain/discomfort, anxiety/depression). Each dimension is scored by choosing one of three responses. The responses recorded are based on levels of severity (no problems/some or moderate problems/extreme problems). Utility scores will be calculated for each mother at each time point based on the algorithm developed by Dolan [67]. Utility scores at each time point will be used to calculate total quality-adjusted life years (QALYs) for the duration of the trial calculated as the area under the curve adjusting for baseline. It will be administered at baseline, 1- and 2-year follow-ups.

*Treatment Experience Questionnaire* (TEQ). This is a 15-item feedback questionnaire based on questionnaires used for similar studies (e.g. [68, 69]. This will be given to participants in the MTB arm of the trial only, to record satisfaction with the service they have received. Parents are asked to rate the treatment on a 5-point scale (disagreement to agreement). It will be administered at the year 1 and year 2.

*Father outcome measures*. Where possible we aim to collect selected outcome measurements from fathers at baseline, year-1 and year-2 follow-ups. Some of the outcome measures used for the mothers will also be used for the fathers: quality of life (i.e. EQ-5D); mental health (i.e. EPDS, STAI and PCL-5), support and personal network (i.e. NSSQ), and paternal competence (i.e. SM and PSI), and the Treatment Experience Questionnaire (TEQ) for fathers in the MTB group. In Table 1 mother and child outcome measures are summarised and the time points of their administration reported.

**Table 1:** Outcome measures: description and validity of measures as well as time points of their administration

Outcome measures	Description of and validity of measures	Time points
Primary outcomes		
Maternal sensitivity	Emotional Availability Scales (EA). Observation of behaviours. Score 6 dimensions on a 1 to 7 scale. Validated for international use [79]	Year 1 and year 2
Secondary outcomes		
Child attachment security	Attachment Q-Set (Q-Set). Observation of behaviours. Score on a continuum of secure to insecure. Good convergent and discriminate validity [40]	Year 2
Child cognitive and language development	Bayley Scales of Infant and Toddler Development Scales, third edition (Bayley-III). Individual administration. Continuous scales produce scores. Validated for UK and Ireland use [43]	Year 2
Behavioural problems	Child Behaviour Checklist (CBCL).100-item questionnaire. Responses are on a scale of 0 to 2. Validated for international use [45]	Year 2
Postponed child-bearing	Mother asked about her pregnancy status. Number of months from baseline to the next pregnancy used for analyses. Extensive use with similar studies (e.g. [1])	6 months, year 1, 18 months and year 2
Maternal mental health	Edinburgh Postnatal Depression (EPDS).10-item questionnaire. Responses are on a scale of 0 to 3. Validated measure of depression [47]	Baseline, year 1 and year 2
Child quality of life (QoL)	Warwick Child Health and Morbidity Profile (WCHMP). 10-items survey. An incremental cost-effectiveness ratio (ICER) calculated. Validated with low interobserver variation [49]	Year 1 and year 2
Health and social care resource use	Service Use and Support (SUS). 36-item questionnaire. Cost of services calculated with the Personal Social Services Research Unit (PSSRU). Extensive use in clinical studies (e.g. [80])	Baseline, 6 months, year 1, 18 months and year 2
Additional outcome measures		
Measurement of temperament	Infant Behaviour Questionnaire Revised (IBQ-R). 37-item questionnaire. Responses are on a scale of 1 to 7. Good internal consistency reliability and convergent validity [52]	Year 1
Sensitivity scale	Maternal and paternal Sense of Mastery (MSM). 7-item questionnaire. Responses are on a 7-item scale (agreement to disagreement). Extensive use with similar sample of young women [53]	Baseline, year 1 and year 2
Social support	Norbeck Social Support questionnaire (NSSQ). 9-item questionnaire. Responses are on a scale of 0 to 4. Validity and reliability on all measures [57]	Baseline, year 1 and year 2
Infant health outcome	Health records reviewed at the end of the study and data collected on different issues, including hospitalisation and Social Services’ referrals. Extensive use with similar studies (e.g. [1])	Year 1 and year 2
Parental representation of their child	Parent Development Interview – Revised (PDI). 20-item interview. Scores are on a scale of 1 to 9. Validity shows links to adult attachment and child attachment [29, 32, 58, 59]	Year 1
Stress within the parenting role	Parental Stress Inventory Short Form (PSI-SF). 36-item questionnaire. Responses are on a 5-point scale (agreement to disagreement). Short Forms show concurrent validity with the full-length PSI [62]	Year 1 and year 2
PTSD Checklist Civilian	Posttraumatic stress disorder (PCL-5). 20-item questionnaire Responses are on a scale of 0 to 4. PCL-5 has good psychometric properties [64]	Baseline, year 1 and year 2
State and trait anxiety	State-Trait Anxiety Inventory (STAI). 40-item questionnaire. Responses are on a scale of 0 to 4. Strong construct and concurrent validity [65, 66]	Baseline, year 1 and year 2
Adult quality of life (QoL)	EuroQol EQ-5D 3 level (EQ-5D-3 L) 6-item questionnaire. Responses are on a scale of 0 to 2. Extensive use for similar study (e.g. [81, 82])	Baseline, year 1 and year 2
Treatment experience	Treatment Experience Questionnaire (TEQ). 15-item questionnaire. Responses are on a 5-point scale. Based on questionnaires used in similar studies [68]	Year 1 and year 2

### Sample size

A minimum of 200 participants (100 in each arm) will enter into the evaluation. The sample size calculation is motivated by the effect size estimates on the primary outcome (maternal sensitivity) and the attachment outcome at 1 year.

#### Power analysis

We based our power analyses on previous interventions aimed at improving parenting sensitivity. The overall meta-analytic average for sensitivity-focussed intervention trials in Bakermans-Kranenburg’s (2003) review was *d* = .44 which is equivalent to a correlation of *r* = .22. If we assume four covariates and a single df test of treatment effect, with a reduced model R^2^ of .15 and a full model R^2^ of .20, then 129 participants would be required for 80 % power at alpha = .05. Bakermans-Kranenburg further reported that the meta-analytic average of randomised studies was *d* = .36 (*r* = .18), which for the equivalent analysis and power would require a sample size of 190. We also computed power to detect effects on attachment security. We estimated the effect size based on meta-analytic data, based on the assumption that the MTB intervention would be effective in enhancing parental sensitivity: such studies yield average effect sizes of *d* = .45 in the aforementioned meta-analysis [70] and hence the power for this outcome would be equivalent or greater to that for sensitivity.

### Recruitment

Recruitment will take place at three UK sites: York, Sheffield and Glasgow. Participants in York and Sheffield will be screened if they live within a defined geographical area around each site of approximately 15 miles of the city centre (the precise geographical boundaries will vary in each site).

### Consent

#### Overview

Formal consent into this study will be taken by a member of the research team. Prior to this, consent to be contacted by the research team will be obtained by research midwives in antenatal clinics, by health, social care or voluntary sector professionals or provided by interested families directly.

### Consenting procedures

#### Primary entry-point into the study

At all three sites potentially eligible expectant mothers will be informed about the MTB Study during an antenatal appointment in the hospital or in the community. During this appointment expectant mothers will be given a Participant Information Sheet and a contact leaflet and a research midwife or member of the antenatal care team will provide a brief explanation of the study. Potential participants will then be followed up by a research midwife who will check eligibility, provide them with written information about the study again (Participant Information Sheet and a ‘contact leaflet’) and will verbally explain their involvement. This will usually be done in person at the 20-week scan appointment, but may also be done by telephone (with written material sent by post) or during another antenatal appointment. If expectant mothers are then happy to consent to be contacted by the research team, this will be obtained verbally, and formal written consent to participation in the study will be obtained by the research team during an initial home visit.

During the research home visit the researcher will explain the study in detail, answer any further questions they might have, and, if they are willing to take part, obtain their full written consent. At this research appointment baseline assessments will be carried out for all consenting participants.

### Alternative entry-points into the study

At all three sites, posters, contact leaflets and Patient Information Sheets will be placed in antenatal waiting rooms so that expectant parents can read about the study while they wait for their antenatal appointment. Families who are interested in taking part in the study may self-refer by filling in a contact leaflet and leaving it in a designated box which will be provided at the clinic. These forms will then be collected by the research midwives, and passed to the research team who will then get in touch to arrange a visit, following the same informed consent procedures described above. Similar contact leaflets and Participant Information Sheets will also be distributed to community midwives and other health, social care and voluntary-sector professionals (e.g. GPs, local authority housing officers, Shelter) in the area so that if they know of mothers meeting the eligibility criteria they can make them aware of the study. Such mothers would be directed to the research team’s contact telephone number, or contact leaflets can be sent to the research team, who will then call the participant. Professionals working with families, having obtained verbal consent, may also contact the research team on behalf of the family. Once the research team has obtained confirmation of a participant’s wish to be contacted, the research team would then arrange an initial visit, where the expectant mother would be informed about the study, given an opportunity to ask questions and consented in the standard way described above.

#### Sheffield and Glasgow sites

FNP is offered as a clinical service to all mothers under the age of 20 at the Sheffield and Glasgow sites. Both FNP and MTB have similar entry criteria and a similar set of intervention procedures and as such it will not be possible for parents to be involved in both programmes. As mentioned above, participants will be recruited to the MTB trial at their 20-week scanning appointment. Both Sheffield and Glasgow FNPs enroll parents into the programme up until 20 weeks’ gestation and, as such, the MTB trial will not interfere with client accessibility to the FNP treatment. However, participants will be excluded if they are receiving services from the FNP. This criterion is necessary to ensure the integrity of the TAU arm of the trial. Participation in the FNP will be recorded in the mother’s notes, so that the research midwife is able to selectively recruit non-FNP participants.

### Eligibility criteria

1. Inclusion:
  Women expecting their first baby *and*
  Aged 19 years or under *or* aged between 20 to 25 years and any of the following: (1) currently eligible for means-tested benefits (or someone they live with and depend upon, such as a partner or parent, is eligible for means-tested benefits), (2) not entitled to employer maternity pay and (3) living in a postcode falling within the highest quintile of social deprivation as defined by national government statistics or living in sheltered accommodation
2. Exclusion
  Expectant mothers with a psychotic illness
  Expectant mothers with substance abuse disorders/chronic drug dependence
  Expectant mothers with profound or severe learning disabilities
  Expectant mothers who would require the use of an interpreter
  Expectant parents with a life-threatening illness
  Expectant parents whose baby is expected to be born with a life-threatening illness or profound disability
  The expectant mother has been accepted in a FNP service (see ‘Recruitment’ above)

### Scope of consent to participation

Consent Forms signed by the mother will include permission to access health and social care records, remaining in effect for 3 years (with the provision of course that families may withdraw this consent at any time). Ethical issues are discussed in greater depth below, but we note at this point that in addition to obtaining consent to access medical and social care records, the recruiter will be obliged to explicitly explain the limits of confidentiality in the event that a child protection concern arises. For those not consenting to participate, we will nevertheless endeavour to obtain anonymised summary data from primary care services to characterise these cases, as prior work by our group has found that these missing cases over-represent populations in most need [71]. For any families that drop out of the clinical project after randomisation, we will endeavour to retain them in the research study in order to minimise bias. In addition, even families who drop out of the research study will be asked whether permission can remain to access their medical and social care records so that data on child health outcomes can nevertheless be obtained. Those who are allocated to the treatment arm and later decide to withdraw from the research will still be able to receive MTB treatment if they wish to.

### Randomisation

Eligible consenting participants will be randomised on a 1:1 basis by the randomisation centre (supervised by Peter Fonagy) at a separate site, who will manage randomisation. Monitoring of data quality and integrity will be done separately by David Wellsted, study statistician. Together they will act as DMEC and will have power to break confidential ID codes should ethical concerns arise. A computer-generated adaptive minimisation algorithm [72] that incorporates a random element will be used with the following balancing factors: treatment centre, maternal age (below 20 versus 20 years or older) and current depressive symptomatology (< 10 versus ≥ 10 on the EPDS). These minimisation factors have been selected because previous research has shown that these factors are associated with poorer outcomes on some of our dependent measures or are highly plausible treatment modifiers. Once a family has been approached and has consented to take part, anonymised screening data will be sent to the randomisation centre by the trial coordinator. The randomisation centre will send the results of the randomisation to the local clinical manager within 72 h, ensuring that the research team is fully blind to the condition that the family is allocated to. Participants will be informed about their group allocation, as blinding to a psychosocial treatment of this nature is not possible. The outcome assessors will be blind to the participants’ allocation. During training, all research assistants (RAs) will be briefed regarding the importance of blindness to condition and they will record any instances where the participating family discloses its condition inadvertently, so that the impact of this can be examined in the data analysis. Coding of the primary outcome will be done independently from video recordings by raters who have no contact with the participants.

### Planned intervention

#### Minding the Baby®

MTB is a home-visiting programme that helps vulnerable or high-risk first-time mothers aged 14–25. The programme has been developed by the Yale Child Study Centre and the Yale School of Nursing, with the main focus being on the parent-child relationship. The MTB programme is delivered by an interdisciplinary MTB team of highly skilled practitioners, a nurse or health visitor experienced in parental, perinatal and paediatric roles and a social worker or other suitably trained practitioner trained in mental health assessment and intervention.

Mothers are visited weekly at home from the third trimester until the child’s first birthday and then fortnightly until their second birthday. The two MTB practitioners’ visits are alternated weekly. Visits can be increased as required, particularly in times of crisis.

The health practitioner’s role will focus primarily, but not exclusively, on the following:

Parental care and health education

- Practitioners provide ongoing support and information about maternal and infant nutrition and healthy child growth and development, including fetal and postnatal brain development. Support is given regarding the prevention of premature birth and planning for labour and delivery. Practitioners also help pregnant women to begin to anticipate and imagine life with a newborn, what its needs might be, and how one interacts and communicates with a young infant. Practical and educational support is given to women pre and postnatally regarding breast feeding

Child health and development

- The health practitioners undertake routine assessments of the child’s physical, cognitive and social development and provide advice and guidance about the child health, including advice regarding the identification and treatment of illnesses. Practitioners also provide information and advice about a safe environment for the child to reduce incidents of injury. Finally, practitioners provide anticipatory and ongoing guidance about parenting of young infants and toddlers

Mother’s health

- Practitioners are trained to help women think about safe sex and future family planning, provide support and information regarding healthy lifestyles, including smoking cessation support and healthy nutrition and exercise. Practitioners also assist mothers in obtaining support when they experience physical or mental health difficulties (e.g. via primary care) or have ongoing problems with stress

The social/therapeutic role focusses primarily but not exclusively on the following:

Mental health promotion

- Practitioners in this role are trained in psychosocial assessment and will gather a detailed: psychosocial history; explore the mother’s feelings about her pregnancy, her connection to her unborn child, her own history of being raised and her expectations about the parenting role. Practitioners are trained to identify and provide intervention (through direct working or signposting to others services as appropriate) for mental health problems antenatally and postnatally, and are able to provide focussed mother-infant dyadic interaction guidance drawing on principles from parent-infant psychotherapy, and using video feedback to help mothers to attune to the infant’s attachment cues and promote sensitive interactions

Infant/child and family assessment and intervention

- As part of the dyadic work, practitioners also guide mothers in dyadic play and provide developmental guidance, helping to broaden mothers’ repertoire of skills, teaching about typical developmental milestones and facilitating mothers’ creativity in parenting and self-efficacy. Where indicated, the social-therapeutic practitioner will provide couples’ and family counselling and help families to manage the complexities of formal, statutory/legal systems such as housing, disputes around contact, or child protection intervention. The practitioners offer a broad range of support to help families manage crises and provide assistance in supporting the women’s acquiring of key life skills through education and employment

#### Treatment as Usual (TAU)

TAU will be the standard care available in the local community, which will be determined by the needs of each family and the local service provision. The first line of services is provided at the primary care level by universally available professionals such as GPs, health visitors and midwives. For individuals who require more support after birth the help they can receive will vary depending on where they live and the degree of their needs. In general, TAU is often a package of support from family support workers, enhanced health visiting, social worker or midwifery services (listening visits), one-to-one support from clinical psychologists (provided through local CAMHS services), psychotherapists or counsellors, postnatal support groups, crèches providing respite, parenting education workshops, peer-supported groups, home-visiting services, child psychiatry and family therapy. The Service Users and Support (SUS) questionnaire will be used to check what usual care services both groups of participants receive during the trial.

### Intervention fidelity

Adherence to the MTB intervention protocol will be achieved in close collaboration with the Yale team (including the primary developers) in the following ways:

1. All participant contact will be guided by the written intervention manual as well as other training materials
2. All clinicians will receive extensive training in the MTB model via in-person, taped, or videoconference training sessions led by the Yale MTB trainers. The Yale MTB trainers include senior nurse and mental health supervisors and home visitors
3. All MTB practitioners will record detailed information regarding their direct and indirect contact with families
4. In order to ensure that home visits adhere to the Yale MTB intervention programme after each visit practitioners will complete a Home Visit Form. This aims to describe the visits in detail and compare them with the US MTB intervention home visits. In particular practitioners record the length, nature and focus of the visit and the families’ level of engagement. It also summarises the focus of the visit, e.g. parenting, health, mental health, etc., and the time spent on each topic
5. Specially trained supervisors will undertake model fidelity monitoring by random sampling of families at each site and discussing the outcomes with the relevant sites at compliance visits
6. All practitioners receive regular supervision by Yale-trained local UK supervisors. These specially trained supervisors meet monthly via phone with the Yale MTB trainers
7. Regular disciplinary and interdisciplinary supervision will be provided by specially trained supervisors and the Yale MTB team (in addition to supervision provided as usual by the practitioners’ line managers)

### Participant retention

Dropping out of treatment is common in prevention studies in the perinatal period [73]. In one of the key studies of the NFP programme, active refusals to participate in the trial ran at approximately 20 % (with a further 20 % passively dropping out by not responding to mailed invitations to participate), which is higher than the estimates from the Yale pilot study [23]. However, it is notable that a much smaller proportion refused to participate in the research evaluation once they had agreed to randomisation (3.8 %). From the outset of the FNP study to the 2-year outcome phase a further 21 % were lost to follow-up. In the UK, the FNP programme had an initial uptake rate of 83 % of eligible families and a later dropout rate of 15 %. We thus aim to over-recruit by 15 % to take attrition into account, leading to an initial intake target of *N* = 240, so that 100 per arm is achieved at the year-2 outcome point. An overview of participant timeline is presented in Table 2.

**Table 2:** Time requirement per participant

		Study period				
			Post allocation			
Time point	Prebaseline	Baseline	6 months	Year 1	18 months	Year 2
Recruitment:						
Eligibility screen	X					
Informed consent	X					
Allocation		X				
Research assessment:						
Questionnaires		X	X	X	X	X
Reflective Functioning				X		
Maternal sensitivity				X		X
Developmental assessment						X
Attachment classification				X		X
Overall time involvement	15 min	1 h	15 min	2 h	15 min	2 h

### Data management

The data will be collected by experienced RAs who have been trained to work with high-risk populations. Necessary safeguarding policies will be in place to ensure the safety of the RA collecting the data. In particular, contact information of the assessment location will be left with another member of staff before leaving for the assessment. Regular contact with the RA will be maintained at the start and end of the assessment. In situations where an RA feels to be in immediate danger they will be instructed to follow safeguarding policies to call the police.

Regular supervision with the trial management team, the coordinator and the principal investigators will ensure the reliability of data collection. Where necessary the RAs will be fully trained and certified in administering and coding research measures.

All coding will be supervised by the principal investigators. Where standardised coding measures are required the RAs will undertake full training courses and complete necessary reliability checks. The data will be coded by an RA who does not know the family and will be blind to the subject status (intervention or control). Interrater reliability will be established for all instruments.

Every week, questionnaire data collected the previous week will be coded, verified and double-entered directly into secure web databases. Audio interviews will be transcribed and video-taped material downloaded, any personal identifiable information will be removed and the data stored on a secure server ready for coding. To check the reliability of the process, 10 % of the records will be randomly selected and will be reviewed, coded and entered independently by RAs for calculation of interrater agreement rates. The databases will be compared and checked for errors before transferring to an SPSS (v. 21.0) file for analysis.

### Data transfer

In the study, all participant data as outlined previously in this protocol will be collected in accordance with the participant Consent Form and Participant Information Sheet. All participant data will be appropriately sent to Dr. David Wellsted for statistical analysis and UCL will act as the data controller of such data for the study. Professor Pasco Fearon will be responsible for the processing, storage and disposal of all participant data in accordance with all applicable legal and regulatory requirements, including the Data Protection Act 1998 and any amendments thereto.

Data will be stored on a secure server dedicated exclusively to this project that has encrypted access. Only the research team will have access to the data and to information identifying participants. Research data and personally identifying data will be stored in separate, web-accessible, secure databases. All research data will be stored in locked filing cabinets in each site. Similarly, Consent Forms will be stored separately from the research data in locked filing cabinets in each site. Risks to subject confidentiality will be minimised by adopting suitable data storage procedures in accordance with best practice guidelines and in accordance with the Data Protection Act. Subjects will be assigned ID numbers. The master ID list that links subject names with ID numbers will be kept on a highly secure password-protected server. All information concerning allocation to condition (TAU or MTB) will be held securely by the randomisation centre. Clinical records and other relevant clinical information regarding participants in the MTB arm will be held by the NSPCC, following their standard governance protocols.

### Data analysis

The primary outcome, maternal sensitivity, is an average of several ordinal scores and is typically found to be approximately normally distributed. The primary analysis will be a regression analysis testing group differences in mean sensitivity at year 1 after adjustment for baseline characteristics. Clustering by therapist and site will be allowed for by computing robust standard errors [74]. Continuously distributed secondary outcomes will be treated in the same manner. The risk of child protection intervention will be described using the Kaplan-Meier method and summarised by the proportions of children with child protection intervention over 2 years period of observation. The primary analysis for this outcome will be Cox regression, adjusting for key baseline characteristics.

Where there are missing data, these will be evaluated either by multiple imputation or a sensitivity analysis determined by the pattern of missing data. In doing so, we will follow the procedures and guidance outlined by Sterne and colleagues [75]. Mediational analyses of change mechanisms (e.g. age 12 months’ maternal sensitivity-mediating treatment effects on attachment at age 2 years) will be tested using bootstrap methods described by MacKinnon and Dwyer [76] and Preacher and Hayes [77].

### Additional data analysis

#### Economic evaluation

We will conduct a cost-effectiveness analysis of MTB relative to the control condition from a broad societal perspective.

Cost information: we propose two elements to the cost component of the cost-effectiveness analysis:

1. Cost of MTB: this will include fixed costs associated with the resources required to run the service as well as variable costs associated with training, staffing and related consumables. We will calculate a bottom-up costing of the service and calculate a weighted cost per case based on the caseload of each practitioner
2. Costs of the use of other resources: we will use a self-completed Service User and Support (SUS) questionnaire to collect other health and social care and out-of-pocket costs for clients in the MTB and the control group. The retrospective self-completed questionnaire will provide information on resources accessed during the last 6 months. The SUS will be completed at enrolment, 6 months after the baby is born by telephone and at each outcome assessment (infant age 1 and 2 years). Resource use will be costed using Personal Social Services Research Unit (PSSRU) and national datasets wherever possible

We will provide summary statistics of the costs for the MTB and the control group as well as a comparison of the total cost per patient to society of MTB compared to controls for the duration of the study.

Incremental cost-effectiveness ratio (ICER): the ICER is the mean cost per mother/child in the intervention minus the mean cost per patient in the control group divided by the mean incremental gain per mother/child in outcomes from the intervention compared to the controls. If an intervention has a lower cost to society and better outcomes it is considered dominant and likely to be adopted by a decision-maker if the evidence is satisfactory. If the intervention has higher cost to society but is associated with better outcomes the decision-maker needs adequate information to determine if they are willing to pay the additional cost per outcome gained.

We propose calculating a number of ICERs for MTB compared to controls and propose using the following outcomes in the denominator of the ICER for different analyses:

- Maternal sensitivity
- Infant QoL using the Warwick Child Health and Morbidity Profile [20, 78]
- Parental QoL using the EQ-5D, which is a brief questionnaire that measures generic health-related quality of life from the patient’s point of view. EQ-5D scores can be converted to preference-based utility scores that can be used to calculate quality-adjusted life years (QALYs) for use in cost-effectiveness analyses using an algorithm developed by Dolan [67]
- Mother-infant attachment

As the ICER does not easily allow for normal statistical tests we will use bootstrapping methods, replications of the statistic of interest by sampling with replacement from the original data, to calculate the confidence interval for the ICER. We will also use this data and the net-monetary benefit approach to calculate the probability that MTB is cost-effective compared to the control group for a number of values of willingness to pay per gain in outcome or the cost-effectiveness acceptability curve (CEAC) [19]. This provides more information to decision-makers to help them decide if the outcomes achieved as a result of the intervention are worth the additional cost.

Lifetime model: poor parent-child relationships, child abuse and neglect can have long-term negative impacts on children, their families and society. Poor parenting has repeatedly been identified as being associated with antisocial behaviour and severe behavioural problems [22, 23]. A long-term follow-up study of children with conduct disorder suggested that the cost of unresolved conduct disorders can exceed £1 million over an individual’s lifetime [2]. There are obviously further costs and benefits to realise as a result of preventing each case of child abuse and neglect. The ICERs proposed above do not capture the full lifetime costs and outcomes that may be realised as a result of MTB. As part of the project we would, therefore, aim to investigate developing a decision analytical model that uses information available from the evaluation, as well as published data sources, to determine the cost-effectiveness of MTB over the lifetime of the children.

### Data monitoring

#### Data monitoring

The Trial Steering Committee (TSC) will take the role of monitoring trial safety and data monitoring. The statistician will review the data on an ongoing basis, including any adverse event records, and report this to the TSC. Detailed reports will be prepared by the TSC statistician to monitor safety/adverse event data, recruitment and dropout rates. The formal statistical interim analysis of the primary outcome will be reported to the TSC after the end of the first outcome phase.

#### Trial Steering Committee

A TSC will be used to monitor the progress of the project and advise the research team on matters arising during subsequent phases of the study. The TSC will meet 6-monthly and perhaps more regularly during the preparatory and final stages of the formal evaluation. The group will be made up of representatives from the NSPCC, researchers, a statistician, service users and/or carers and representatives of professional/provider organisations, including a link person from at least two local clinical teams.

### Ethical considerations

This trial has received a multisite ethics approval from the NHS Health Research Authority (NRES) Research Ethics Committee (London-Dulwich, the United Kingdom) (REC reference: 13/LO/1651; IRAS project ID: 135643; protocol version 6.0, 11 January 2016). Research and Development (R&D) approval is in place at all three sites. A formal amendment is needed for any modification of the protocol and requires approval by the NHS REC as well as the local R&D office’s approval.

## Discussion

The study protocol presented in this paper explains how MTB, a programme aimed to support young, vulnerable first-time parents with their baby, will be evaluated in a randomised trial in the UK. A key feature of this approach is the way in which it combines health input from community nurses with mental health input from social workers. Another key feature is the explicit focus on promoting sensitivity of parenting, and a model of change based on the assumption, supported by developmental research, that parental RF is critical in promoting sensitive and attuned interactions between mother and infant. The trial represents the first UK study of MTB.

The MTB programme was developed at Yale University where a pilot trial produced encouraging results [1]. Positive outcomes emerged in relation to attachment as well as health and mental health outcomes. In particular, infants allocated to the MTB group showed higher rates of secure attachment, and mothers showed improvements in maternal RF as well as positive health outcomes compared to the control group. Crucially, these outcomes appeared to be lasting as benefits continued to be observed when the children were seen at the ages of 3 and 5 years.

We predict that similar outcomes will emerge from this intervention in the UK. In particular, mothers randomised to the MTB group, compared to the mothers in the TAU group, are expected to show higher observed sensitivity as well as more secure attachment. Findings will be published in scientific journals, shared with stakeholders and will inform child and maternal health policy. The study will have important implications for the delivery of early intervention to families who are potentially at risk, especially during the crucial first months and years of life, from pregnancy to age 2 years.

### Trial status

Recruiting of expectant mothers started in April 2014 and we are still recruiting.

## Additional file

Additional file 1: The SPIRIT checklist. (DOC 123 kb)

## Abbreviations

CAMHS: Child and Adolescent Mental Health Services
CBCL: Child Behaviour Check List
CEAC: Cost Effectiveness Acceptability Curve
DMEC: Data Management and Ethics Committee
EPDS: Edinburgh Postnatal Depression Scale
EQ-5D: EuroQol EQ-5D 3 level
IBQ-R: Infant Behaviour Questionnaire Revised
ICER: Incremental cost-effectiveness ratio
MSM: Maternal Sense of Mastery
MTB: Minding the Baby®
NFP: Nurse Family Partnership
NRES: National Research Ethics Services
NSPCC: National Society for the Prevention of Cruelty to Children
NSSQ: Norbeck Social Support Questionnaire
PSI: Parenting Stress Index
PTSD: Post-Traumatic Stress Disorder
RF: Reflective Functioning
STAI: Spielberger State-Trait Anxiety questionnaire
SUS: Service Use and Support Questionnaire
TEQ: Treatment Experience Questionnaire

## References

[1] Sadler LS, Slade A, Close N, Webb DL, Simpson T, Fennnie K, Mayer LC (2013). Minding the Baby: enhancing reflectiveness to improve early health and relationship outcomes in an interdisciplinary home visiting program. Infant Mental Health J.

[2] Olds D, Henderson CR, Kitzman HJ, Enckerode JJ, Cole RE, Tatelbaum RC (1999). Prenatal and infancy home visitation by nurses: recent findings. Future Child.

[3] Olds D, Kitzman H, Cole R, Robinson JA (1997). Theoretical foundations of a program of home visitation for pregnant women and parents of young children. J Community Psychol.

[4] Slade A (2005). Parental reflective functioning: an introduction. Attach Hum Dev.

[5] Goldberg S (2000). Attachment and development.

[6] Ordway M, Sadler LS, Dixon J, Close N, Mayes L, Slade A (2014). Lasting effects of an interdisciplinary home visiting program on child behavior: preliminary follow-up results of a randomized trial. J Pediatr Nurs.

[7] Ermisch J (2003). Does a ‘teen-birth’ have longer-term impacts on the mother? Suggestive evidence from the British Household Panel Study. IDEAS Working Papers Series of the Institute for Social and Economic Research, paper 2003–32.

[8] Swann C, Bowe K, McCormick G, Kosmin M (2003). Teenage pregnancy and parenthood: a review of reviews Great Britain: NHS Health Development Agency; National Institute for Health and Clinical Excellence.

[9] Lesser J, Koniak-Griffin D (2000). The impact of physical or sexual abuse on chronic depression in adolescent mothers. J Pediatr Nurs.

[10] Pearlin L, Menaghan EG, Lieberman MA, Mullan JT (1981). The stress process. J Health Soc Behav.

[11] Sadler L, Anderson SA, Sabatelli RM (2001). Parental competence among African American adolescent mothers and grandmothers. J Pediatr Nurs.

[12] Sadler LS, Swartz MK, Ryan-Krause P (2003). Supporting adolescent mothers and their children through a high school-based child care center and parent support program. J Pediatr Health Care.

[13] Singh G, Ghandour RM (2012). Impact of neighborhood social conditions and household socioeconomic status on behavioral problems among US children. Matern Child Health J.

[14] Brooks-Gunn J, Duncan GJ (1997). The effects of poverty on children. Future Child.

[15] Sellström E, Bremberg S (2006). The significance of neighbourhood context to child and adolescent health and well-being: a systematic review of multilevel studies. Scand J Public Health.

[16] Olds D, Robinson J, O’Brien R, Luckey DW, Pettitt LM, Henderson CR (2002). Home visiting by paraprofessionals and by nurses: a randomized, controlled trial. Pediatrics.

[17] Kitzman H, Olds DL, Henderson CR, Hanks C, Cole R, Tatelbaum R (1997). Effect of prenatal and infancy home visitation by nurses on pregnancy outcomes, childhood injuries, and repeated childbearing: a randomized controlled trial. JAMA..

[18] Kitzman H, Olds DL, Sidora K, Henderson CR, Hanks C, Cole R (2000). Enduring effects of nurse home visitation on maternal life course: a 3 year follow up of a randomised trial. JAMA.

[19] Olds D, Henderson CR, Phelps C, Kitzman H, Hanks C (1993). Effects of prenatal and infancy nurse home visitation on government spending. Med Care.

[20] Olds D, Henderson CR, Cole R, Eckenrode J, Kitzman H, Luckey D (1998). Long-term effects of nurse home visitation on children’s criminal and antisocial behaviour: 15 year follow-up of a randomised controlled trial. JAMA.

[21] Olds D, Hill P, Robinson J, Song N, Little C (2000). Update on home visiting for pregnant women and parents of young children. Curr Probl Pediatr.

[22] Olds D, Kitzman HJ (1993). Review of research on home visiting for pregnant women and parents of young children. Future Child.

[23] Olds D (2002). Prenatal and infancy home visiting by nurses: from randomized trials to community replication. Prev Sci.

[24] Lieberman A, Weston DR, Pawl JH (1991). Preventive intervention and outcome with anxiously attached dyads. Child Dev.

[25] Cassidy J, Shaver PR (2008). Handbook of attachment: theory, research, and clinical applications.

[26] Ainsworth MDS, Blehar MC, Waters E, Wall S (1978). Patterns of attachment: a psychological study of the strange situation.

[27] Main M, Kaplan N, Cassidy J (1985). Security in infancy, childhood, and adulthood: a move to the level of representation. Monogr Soc Res Child Dev.

[28] De Wolff M, van Ijzendoorn MH (1997). Sensitivity and attachment: a meta-analysis on parental antecedents of infant attachment. Child Dev.

[29] Slade A, Belsky J, Aber JL, Phelps JL (1999). Mothers’ representations of their relationships with their toddlers: links to adult attachment and observed mothering. Dev Psychol.

[30] Slade A, Grienenberger J, Bernbach E, Levy D, Locker A (2005). Maternal reflective functioning, attachment, and the transmission gap: a preliminary study. Attach Hum Dev.

[31] Fonagy P, Bateman A, Bateman A (2011). The widening scope of mentalizing: a discussion. Psychology and Psychotherapy. Psychol Psychother Theory Res Pract.

[32] Grienenberger J, Kelly K, Slade A (2005). Maternal reflective functioning, mother-infant affective communication, and infant attachment: exploring the link between mental states and observed caregiving behavior in the intergenerational transmission of attachment. Attach Hum Dev.

[33] Moher DSK, Altman DG (2001). The CONSORT statement: revised recommendations for improving the quality of reports of parallel-group randomized trials. Ann Intern Med..

[34] Chan A-W TJ, Altman DG, Laupacis A, Gotzsche PC (2013). SPIRIT 2013 Statement: defining standard protocol items for clinical trials. Ann Intern Med..

[35] Belsky J, Fearon RMP, Bell B (2007). Parenting, attention and externalizing problems: testing mediation longitudinally, repeatedly and reciprocally. J Child Psychol Psychiatry.

[36] Smith P, Pederson DR (1988). Maternal sensitivity and patterns of infant-mother attachment. Child Dev.

[37] McElwain N, Booth-LaForce C (2006). Maternal sensitivity to infant distress and nondistress as predictors of infant-mother attachment security. J Fam Psychol.

[38] Biringen Z, Robinson JL, Emde RN (2000). Appendix B: the Emotional Availability Scales (3rd ed.; an abridge Infancy/Early Childhood Version). Attach Hum Dev..

[39] Waters E (1995). The Attachment Q-Set. Monogr Soc Res Child Dev.

[40] van Ijzendoorn M, Vereijken CM, Bakermans-Kranenburg MJ, Riksen-Walraven JM (2004). Assessing attachment security with the Attachment Q Sort: meta-analytic evidence for the validity of the observer AQS. Child Dev.

[41] Fearon R, Bakermans-Kranenburg MJ, van Ijzendoorn MH, Lapsley AM, Roisman GI (2010). The significance of insecure attachment and disorganization in the development of children’s externalizing behavior: a meta‐analytic study. Child Dev.

[42] Bayley N (2006). Bayley Scales of Infant and Toddler Development.

[43] Marlow N, Wolke D, Bracewell MA, Samara M (2005). Neurologic and developmental disability at six years of age after extremely preterm birth. N Engl J Med..

[44] Achenbach T (1991). Manual for the Child Behavior Checklist/4–18 and 1991 Profile.

[45] Ivanova M, Achenbach TM, Rescorla LA, Harder VS, Ang RP, Bilenberg N (2010). Preschool psychopathology reported by parents in 23 societies: testing the seven-syndrome model of the child behavior checklist for ages 1.5–5.. J Am Acad Child Adolesc Psychiatry.

[46] Cox J, Holden J, Sagovsky R (1987). Detection of postnatal depression. Development of the 10-item Edinburgh Postnatal Depression Scale. Br J Psychiatry.

[47] Cox J, Chapman G, Murray D, Jones P (1996). Validation of the Edinburgh Postnatal Depression Scale (EPDS) in non-postnatal women. J Affect Disord.

[48] Murray D, Cox JL (1990). Screening for depression during pregnancy with the Edinburgh Depression Scale (EDDS). J Reprod Infant Psychol.

[49] Coe C (1996). The development and validation of a measure off parent‐reported child health and morbidity: the Warwick Child Health and Morbidity Profile. Child Care Health Dev.

[50] Byford S, Harrington R, Torgerson D, Kerfoot M, Dyer E, Harrington V (1999). Cost-effectiveness analysis of a home-based social work intervention for children and adolescents who have deliberately poisoned themselves. Results of a randomised controlled trial. Br J Psychiatry.

[51] Kohnstamm G, Bates J, Rothbart M (1989). Temperament in childhood.

[52] Parade S, Leerkes EM (2008). The reliability and validity of the Infant Behavior Questionnaire-Revised. Infant Behav Dev.

[53] Pearlin L, Schooler C (1978). The structure of coping. J Health Soc Behav.

[54] DeSocio J, Kitzman H, Cole R (2003). Testing the relationship between self‐agency and enactment of health behavior. Res Nurs Health.

[55] Norbeck J, Lindsey AM, Carrieri VL (1981). The development of an instrument to measure social support. Nurs Res.

[56] Norbeck J, Lindsey AM, Carrieri VL (1983). Further development of the Norbeck Social Support Questionnaire: normative data and validity testing. Nurs Res.

[57] Gigliotti E (2002). A confirmation of the factor structure of the Norbeck Social Support Questionnaire. Nurs Res.

[58] Levy DW, Truman S (2002). Reflective functioning as mediator between drug use, parenting stress and child behaviour.

[59] Slade A (1999). Representation, symbolization and affect regulation in the concomitant treatment of a mother and child: attachment theory and child psychotherapy. Psychoanal Inq..

[60] Aber J, Belsky J, Slade A, Crnic K (1999). Stability and change in mothers’ representations of their relationship with their toddlers. Dev Psychol.

[61] Abidin R (1995). Parenting Stress Index, third edition: professional manual.

[62] Abidin R (1983). Parenting Stress Index (PSI) Manual, Administration Booklet, and Research Update.

[63] Weathers F, Litz B, Herman D, Huska J, Keane T (1993). The PTSD Checklist (PCL): reliability, validity, and diagnostic utility.

[64] Ruggiero K, Del Ben K, Scotti JR, Rabalais AE (2003). Psychometric properties of the PTSD Checklist—Civilian version. J Trauma Stress.

[65] Spielberger CD, Gorsuch RL, Lushene R, Vagg PR, Jacobs GA (1983). Manual for the State-Trait Anxiety Inventory.

[66] Spielberger CD (1989). State-Trait Anxiety Inventory: a comprehensive bibliography.

[67] Dolan P (1997). Modeling valuations for EuroQol health states. Med Care.

[68] Cooper P, Murray L, Wilson A, Romaniuk H (2003). Controlled trial of the short and long term effect of psychological treatment of postpartum depression. Br J Psychiatry.

[69] Silove D, Parker G, Manicavasagar V (1990). Perceptions of general and specific therapist behaviors. J Nerv Ment Dis..

[70] Bakermans-Kranenburg M, IJ Zendoorn MH, van Juffer F (2003). Less is more: meta-analyses of sensitivity and attachment interventions in early childhood. Psychol Bull.

[71] Murray L, Cooper P, Hipwell A (2003). Mental health of parents caring for infants. Arch Womens Ment Health.

[72] Pocock SJ, Simon R (1975). Sequential Treatment Assignment with balancing for prognostic factors in the controlled clinical trial. Biometrics.

[73] Brugha T, Wheatley S, Taub NA, Culverwell A, Friedman T, Kirwan P, Jones DR, Shapiro DA (2000). Pragmatic randomized trial of antenatal intervention to prevent post-natal depression by reducing psychosocial risk factors. Psychol Med.

[74] Roberts C (1999). The implication of variation in outcome between health professionals for the design and analysis of randomised controlled trials. Stat Med..

[75] Sterne JA, White IR, Carlin JB, Spratt M, Royston P, Kenward MG (2009). Multiple imputation for missing data in epidemiological and clinical research: potential and pitfalls. BMJ..

[76] MacKinnon D, Dwyer JH (1993). Estimating mediated effects in prevention studies. Eval Rev.

[77] Preacher K, Hayes AF (2008). Asymptotic and resampling strategies for assessing and comparing indirect effects in multiple mediator models. Behav Res Methods.

[78] McIntosh E, Clarke P, Frew E, Louviere J (2010). Applied methods of cost-benefit analysis in health care. Handbooks in health economic evaluation series.

[79] Della Jean D (2012). Reliability and validity of the Emotional Availability Scale among Hispanic and African American mother-toddler dyads.

[80] Belfer ML (2008). Child and adolescent mental disorders: the magnitude of the problem across the globe. J Child Psychol Psychiatry.

[81] Wailoo A, Davis S, Tosh J (2010). The incorporation of health benefits in cost utility analysis using the EQ-5D. School of Health and Related Research, University of Sheffield.

[82] Guide to the methods of technology appraisal 2013. NICE. 2013. In: National Institute for Health and Care Excellence. 2013. https://www.nice.org.uk/process/pmg9/. 27905712

